# Biosensors for Seafood Safety Control—A Review

**DOI:** 10.3390/mi15121509

**Published:** 2024-12-18

**Authors:** Thi Ngoc Diep Trinh, Hanh An Nguyen, Nguyen Pham Anh Thi, Nguyen Nhat Nam, Nguyen Khoi Song Tran, Kieu The Loan Trinh

**Affiliations:** 1 School of Engineering & Technology, Tra Vinh University, Tra Vinh City 87000, Vietnam; 2 Institute of Food and Biotechnology, Can Tho University, Can Tho City 94000, Vietnam; 3 Applied Biology Center, School of Agriculture and Aquaculture, Tra Vinh University, Tra Vinh City 87000, Vietnam; 4 NTT Hi-Tech Institute, Nguyen Tat Thanh University, Ward 13, District 04, Ho Chi Minh City 70000, Vietnam; 5 BioNano Applications Research Center, Gachon University, 1342 Seongnam-daero, Sujeong-gu, Seongnam-si 13120, Gyeonggi-do, Republic of Korea

**Keywords:** seafood safety, biosensors, electrochemical, colorimetric, SERS

## Abstract

The increased demand for consuming seafood has made seafood production undergo a rapid period of growth. However, seafood has a high risk of contamination from harmful microorganisms and marine toxins which can cause health problems for humans consuming it. Concerning this issue, monitoring seafood safety has become a center of attention for researchers, and developing effective methods for detecting contamination in seafood has become a critical research field. In this context, biosensors have served as a promising approach to monitor seafood contamination. Compared to conventional methods, biosensors have some key benefits such as high sensitivity, selectivity, portability, and user-friendly operation. Along with significant advances in biosensors, processes of seafood monitoring can be simplified and performed outside the laboratory. In this review article, we describe the mechanisms of two main types of biosensors regarding electrochemical and optical biosensors. The current reports within the last five years on the application of these biosensors for seafood monitoring are also summarized.

## 1. Introduction

The demand for seafood consumption has significantly increased, which has made seafood production witness a period of fast growth. However, in natural environments, seafood is easily contaminated by marine biotoxins such as tetrodotoxin, saxitoxin, okadaic acid, domoic acid, brevetoxin-1, and azaspicacid-1 [1,2]. In addition, seafood provides a favorable environment for harmful microorganisms to grow, which can cause severe infectious diseases for humans consuming them. Some common harmful microorganisms found in seafoods are *Vibrio vulnificus*, *Vibrio parahaemolyticus*, *Escherichia coli*, *Staphylococcus aureus*, and *Pseudomonas aeruginosa* [3,4]. In this context, quality and safety issues are critical aspects that need to be seriously concerned because they play an important role in protecting human health and maintaining consumer confidence and trust. Conventional methods for monitoring seafood quality employ some tests that are performed in laboratories equipped with modern equipment. These conventional methods have limited applicability in low-resource settings [5].

Biosensors serve as potential solutions for overcoming the limitations of conventional methods for seafood monitoring. The biosensor is an integrated device which can analyze the analytes through converting biological response into measurable signals such as electrical, optical, or electrochemical signals [6]. In the current decade, biosensors have become the center of attention for scientists due to their significant advantages such as high sensitivity, selectivity, portability, and user-friendly operation [7,8]. There are several types of biosensors. Among them, electrochemical and optical biosensors are the most common biosensors for seafood monitoring. Electrochemical biosensors can be used to sense biochemical reactions and convert them into measurable electrochemical signals [9,10]. Meanwhile, optical biosensors sense the presence of target analytes through optical signal. In optical biosensors, there are two common types including colorimetric and SERS biosensors. Colorimetric biosensors detect the presence of target analytes through the color changing [11,12], while SERS biosensors use Raman scattering to qualify and quantify target analytes [13,14,15].

In seafood production, biomarkers provide information about the spoilage or freshness level, toxicity, foodborne pathogens, ecological pollutants, etc. Common biomarkers for monitoring the freshness of seafood are biogenic amines and pH value. During spoilage, biogenic amines tend to accumulate in seafood, which makes them become potential biomarkers for checking the seafood’s freshness. Moreover, ammonia and other nitrogenous compounds accumulate in seafood and volatilize into the air, resulting in changing pH value of seafood products [16]. Therefore, biogenic amines and pH are the two common biomarkers for monitoring seafood spoilage. In addition, seafood can be easily contaminated with a variety of toxins, especially during algae blooms. Marine toxins such as okadaic acid, saxitoxin, tetrodotoxin, and microcystin-LR can cause serious health problems in humans. Therefore, detecting the presence of these marine toxins is vitally important. In addition, seafood are highly contaminated by foodborne pathogens such as *Vibrio parahaemolyticus*, *Vibrio vulnificus*, *Listeria monocytogenes*, and *Escherichia coli* [17,18].

**Figure 1:**
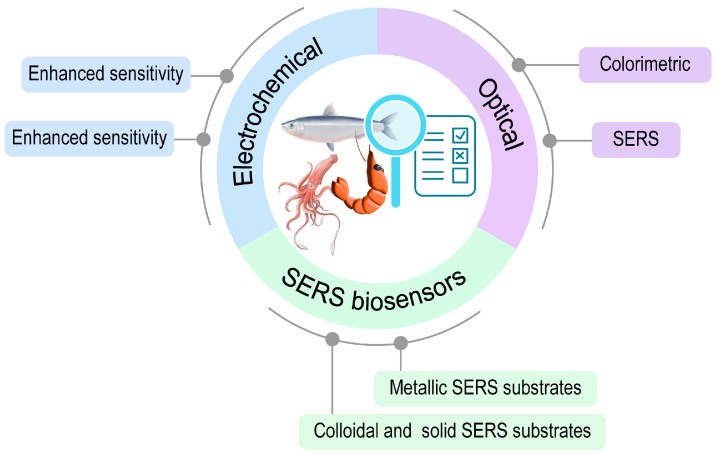
Application of biosensors for seafood monitoring.

Despite the high demand for using biosensors for seafood monitoring, review articles gathering information about this topic are still limited. In this review, we divided biosensors into two main types of biosensors (electrochemical and optical biosensors). The mechanisms, advantages, and disadvantages of each type of biosensor is described. Current reports on biosensors for seafood monitoring are also discussed. Figure 1 shows the summary of this study.

## 2. Common Types of Biosensors for Seafood Monitoring

### 2.1. Electrochemical Biosensors

Electrochemical biosensors are considered one of the most common approaches for seafood analysis due to their outstanding properties such as high sensitivity, specificity, accuracy, and portability. Electrochemical biosensors use electrodes to convert biochemical signals into measurable electrical signals. The working principle of electrochemical biosensors depends on the redox reaction between the immobilized biomolecules on the electrodes and the targeted analytes that generate electron flow. The generated electron flow due to the interaction between target analytes and biomolecules on the electrodes changes the original electrical profile of the solution, indicating the presence of target analytes. Commonly, an electrochemical biosensor comprises three electrodes: working, reference, and counter electrode [19,20]. The working electrode is the most important component because it can transduce biochemical signals into electrical signals [21,22,23]. Meanwhile, counter electrodes enable the electron flow between the working electrode and potentiostat. The reference electrode provides a constant and defined potential and cannot be affected by the composition of the analyte solution. The reference and counter electrodes are mostly constant, while surface modification is strongly required to fabricate the working electrode [24,25,26].

#### Electrochemical Biosensors with Enhanced Specificity and Sensitivity

**Figure 2:**
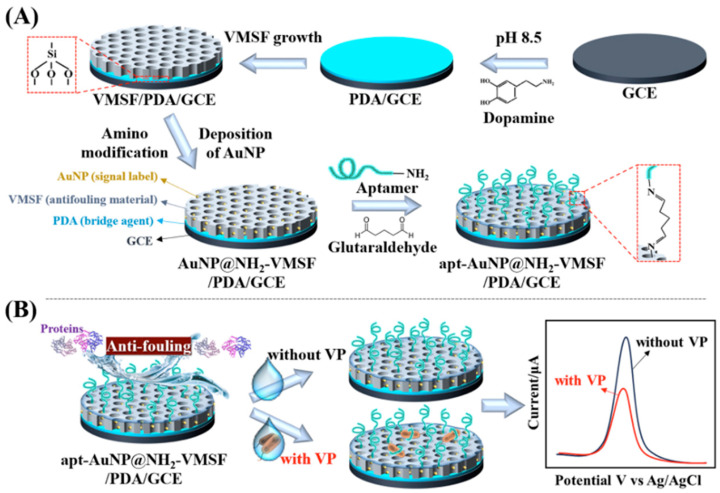
(**A**) Scheme showing the preparation of an aptamer-based electrochemical biosensor. (**B**) Electrochemical detection of *Vibrio parahaemolyticus* apt-AuNP@NH_2_-VMSF/PDA/GCE [28].

For enhancing the specificity of electrochemical biosensors, the surface of electrodes can be coated by biorecognition elements such as aptamers, antibodies, enzymes, and molecularly imprinted polymers. Aptamers are short and single-stranded DNA or RNA that can specifically bind to targets such as proteins, carbohydrates, toxins, cells, and even tissues. Due to the ability to form single-stranded loops and helices, aptamers can generate a variety of shapes. Each shape of aptamer can bind specifically to target analyte. The target recognition involves shape-dependent, three dimensional, hydrophobic, intercalating, base-stacking interactions. For example, Jiang et al. fabricated electrochemical aptasensor through immobilizing anti-*Vibrio parahaemolyticus* aptamer on the surface of the working electrode [27]. This electrochemical aptasensor could distinguish *Vibrio parahaemolyticus* from *Enterococci* and *Escherichia coli* which is the leading cause of seafood-associated bacterial gastroenteritis. Similarly, the electrochemical aptasensor was also confirmed for the ability to detect *Vibrio parahaemolyticus* in complex samples like shrimp homogenate (Figure 2) [28]. This electrochemical aptasensor could distinguish *Vibrio parahaemolyticus* from *Staphylococcus aureus*, *Escherichia coli*, *Pseudomonas aeruginosa*. Alternatively, antibody coating serves as potential options to enhance specificity of electrochemical biosensors. The target recognition principle of antibody relies on the immunoassay in which antibody has high affinity toward antigen of interest. The electrochemical biosensors that apply an immunoassay approach are called electrochemical immunosensors. Electrochemical immunosensors are usually used for detecting biomolecules such as proteins, biotoxins, and antigens. The high specificity of electrochemical immunosensor was confirmed by Nelis et al. through investigating an interference test [29]. In this test, okadaic acid and domoic acid were efficiently distinguished from naturally co-occurring marine toxins: tetrodotoxin and saxitoxin. Enzymes are another important biorecognition element that can improve the specificity of electrochemical biosensors. For example, Sharma et al. immobilized xanthine oxidase on the surface of the working electrode for specifically determining xanthine [30]. The immobilized xanthine oxidase can generate an electrochemical signal through an enzymatic reaction with xanthine. This system successfully detected xanthine in fish samples. In recent decades, molecularly imprinted polymers (MIP) have emerged as potential types of biorecognition element for electrochemical biosensors because they offer simple biosensor preparation and measurement and cost-efficiency. The principle of MIP is imprinting molecular cavities that complementarily fit the target analytes into a synthetic material [31]. For example, Munir et al. immobilized MIP on an electrode surface for detection of histamine which is a toxic metabolite produced during spoilage of fish products [32]. This MIP-based electrochemical biosensor could detect histamine with a linear response ranging from 1 to 1000 nmol/L with 1.765 nmol/L detection limits. Similarly, Hassan et al. synthesized histamine magnetic-MIP by the core–shell method using histamine as a template and 2-vinyl pyridine as functional monomer [33]. The electrochemical biosensor employing histamine magnetic-MIP could detect histamine with detection limit of 1.6 × 10^−6^ mg/L which is much lower than the index for fish spoilage (50 mg/kg^−1^).

During recent decades, along with impressive progress in developing nanotechnology, the sensitivity of electrochemical biosensors can be improved by being coated with nanomaterials. Nanomaterials possess unique properties such as large specific surface area, tailorable pore size, and controllable shape, which can lift the sensitivity of electrochemical biosensors [34,35]. For example, Liang et al. modified a gold electrode with a carboxyl functionalized graphene oxide (GO–COOH) nanocomposite. GO–COOH possessed a large specific surface area (285 m^2^/g) which was higher than that of pristine graphene oxide (100 m^2^/g). Also, GO–COOH had a narrow pore size distribution centered at 3.8 nm, indicating the presence of mesopores. The large surface area and mesopores can facilitate the flow of electrolytes and target analytes resulting in increasing the sensitivity [36]. With the assistance of GO–COOH layer, this electrochemical biosensor possessed a wide linear range from 10 fM to 10 nM for detecting DNA of *Vibrio parahaemolyticus*, with a low detection limit of 3 fM. Sharma et al. coated molybdenum disulfide-molybdenum trioxide (MoS_2_/MoO_3_) onto the surface of electrode [30]. In this system, two-dimensional (2D) MoS_2_ endowed electrodes with unique properties including high surface-to-volume ratio, strong light–matter interaction, and biocompatibility. Meanwhile, MoO_3_ offers advantages such as facilitating electron transfer and increasing stability in aqueous solutions. Taking advantages from MoS_2_/MoO_3_ nanocomposite, this electrochemical biosensor achieved low limit of detection of 64 nM for xanthine detection.

Electrochemical biosensors also have a wide linear range for target analysis. Baskaran et al. demonstrated the practicality of a PDA@ZnMoO_4_/MXene-modified electrode with a linearity of 10–10^7^ CFU/mL for *Listeria monocytogenes* analysis [17]. The electrode modified by PDA@ZnMoO_4_/MXene showed excellent electrochemical properties because of the synergistic effects of MXene, ZnMoO_4_, and PDA. Zheng et al. reported that the linear response of SRCA-CRISPR/Cas12a-based electrochemical biosensor was 5.8 fg/μL–5.8 ng/μL for *Salmonella* detection [37]. For analysis of xanthine in fish, the organic electrochemical transistor-based biosensor had a linear range between 5 and 98 μM [38]. Meanwhile, the electrode modified by MIP exhibited a linear response between 5 μg mL and 25 μg/mL to tetrodotoxin [39].

### 2.2. Optical Biosensors

#### 2.2.1. Colorimetric Biosensors

Colorimetric biosensors serve as one of the most straightforward sensing techniques which rely on the color change in the presence of target analytes. The transduction principle of a colorimetric biosensor is based on the biochemical reaction between a target analyte and a chromogenic material which changes the original color of the solution. The obvious advantage of a colorimetric biosensor is that they can generate naked-eye observable signal output; thus, eliminating bulky equipment in the operating process [40,41,42].

##### Gold Nanoparticles

Gold nanoparticles are one of the most common chromogenic materials used in colorimetric biosensors. Gold nanoparticles emit different colors based on the state of dispersion. When gold nanoparticles are at monodisperse state, the surface plasmon resonance phenomenon causes a green-light absorption resulting in red-light reflection. Meanwhile, at aggregation state, gold nanoparticles change from red to blue [43,44]. Du et al. used a gold nanoparticle-based colorimetric chemosensor array for spoilage monitoring of tuna through a quantitative detection of biogenic amines [45]. In this study, gold nanoparticles were functionalized with three types of carboxylate derivatives (4-mercaptobenzoic acid, 6-mercaptohexanoic acid, and 11-mercaptoundecanoic acid) for detecting ten amine derivatives containing aromatic amines, diamines, and polyamines. Upon the addition of amines, gold nanoparticles responded differently to both amine structures and their concentrations resulting in giving distinct color patterns. The gold nanoparticle-based chemosensor array gave an accurate qualitative and quantitative analysis of ten amines in the mixtures through pattern recognition techniques. Similarly, by changing the surface chemistry through surface modification, gold nanoparticles can be used to selectively detect other target analytes in seafoods. For example, Feng et al. coated truncated aptamers on the surface of gold nanoparticles for detecting microcystin-LR in fish [46]. This aptamer-coated gold nanoparticles can distinguish microcystin-LR from clofentezine, atrazine, and glyphosate with a detection limit of 0.38 ng/mL. Sadri et al. combined gold nanoparticles and magnetic nanoparticles for separation and colorimetric detection of Vibrio parahaemolyticus in raw shrimp samples [47]. Aptamers responding to *Vibrio parahaemolyticus* were conjugated on the surface of both magnetic and gold nanoparticles. Aptamer-magnetic nanoparticles were used as *Vibrio parahaemolyticus* separator, while aptamer-gold nanoparticles were used as colorimetric detector. This combination could distinguish *Vibrio parahaemolyticus* from other bacteria such as *Escherichia coli*, *Salmonella typhimurium*, and *Listeria monocytogenes*. The detection limit was 2.4 CFU/mL.

###### Nanozymes

Alternatively, nanozymes are a promising alternative to the gold nanoparticles approach. Nanozymes have been widely used to fabricate colorimetric biosensors due to their unique properties such as great stability, tailorable surface chemistry, cost-effectiveness, and good biocompatibility. The emergence of nanozymes strongly improved the stability and sensitivity of colorimetric biosensors. Nanozymes comprise artificial nanomaterials that can mimic enzyme activities which can alter the structure of chromogenic materials such as 3,3′,5,5′-tetramethylbenzidine (TMB), resulting in changing TMB color [48,49,50]. For example, Zhang et al. deposited silver nanoparticles (AgNPs) on carbon microspheres (CMs) to form a AgNPs-CMs nanocomplex and used this nanocomplex for detecting Hg^2+^ in seafood [51]. The AgNPs acted as artificial peroxidase nanozyme which can produce superoxide anions (O_2_^•−^) and hydroxyl radicals (•OH). These highly active species strongly triggered the oxidation of TMB and convert TMB into oxidized form with blue color. In the presence of Hg^2+^, the synergistic effect between Hg and Ag significantly enhanced enzyme-mimic activity of AgNPs-CMs. The detection limit of this Hg^2+^ colorimetric test was 1.10 nmol/L.

**Figure 3:**
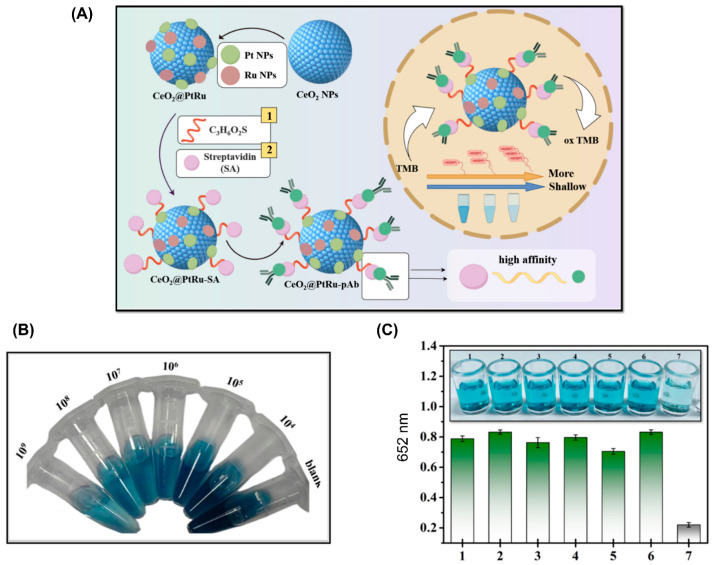
(**A**) Scheme showing the preparation of CeO_2_@PtRu. (**B**) Results showing the colorimetric detection of *Vibrio vulnificus* (10^4^–10^9^ CFU/mL). (**C**) Results showing the selectivity test (1: *Salmonella*, 2: *Vibrio parahaemolyticus*, 3: *Listeria monocytogenes*, 4: *Escherichia coli*, 5: *Bacillus cereus*, 6: sterile PBS, 7: *Vibrio vulnificus*) [52].

The surface properties of nanozymes can be easily designed to fine tune their catalytic performance. Nanozymes have the capability of facile bioconjugation with other molecules providing selective responsiveness to external stimuli. Thus, it is unsurprising to see the current explosion of nanozymes, especially for colorimetric detection. For example, Xu et al. used CeO_2_@PtRu possessing high peroxidase-like activity for constructing a colorimetric biosensor for detecting *Vibrio vulnificus* via immunoassay (Figure 3) [52]. In this study, CeO_2_@PtRu was conjugated with polyclonal antibodies via the biotin-streptavidin system that endowed CeO_2_@PtRu with selective detection of *Vibrio vulnificus*. The binding of *Vibrio vulnificus* onto CeO_2_@PtRu reduced peroxidase-like activity resulting in less production of blue oxTMB. Tian et al. combined Au@Pt nanoparticles and horseradish peroxidase (HRP) to fabricate a dual catalysis system for enhancing the sensitivity of testing okadaic acid [53]. By using dual catalysis, the sensitivity of okadaic acid test was significantly improved with IC_10_ = 0.04 ng/mL, which was 3 or 16-fold more sensitive as compared to Au@HRP or HRP immunoassay, respectively. The detection limit in mussels was 0.6 µg/kg.

The colorimetric biosensors have a wide linear range for target analysis. Lin et al. developed a Cefe-PGA-MNPs and apt-Fe@PDA-based colorimetric biosensor for Vibrio parahaemolyticus detection [54]. This biosensor was demonstrated to possess a broad linear response between 2.1 × 10^1^ and 2.1 × 10^6^ CFU/mL. Li et al. used Fe_7_Ni_3_MOF peroxidase-mimic nanozyme for colorimetric detection of hypoxanthine. The enzymatic cascade system demonstrated a linearity of 3–70 μM with low detection limit of 1.39 μM [55]. Interestingly, Ti_3_C_2_ nanozyme can be porously DNA-encoded (Apt-P-Ti_3_C_2_) via microwave combustion to generate outstanding peroxidase-like activities. The Apt-P-Ti_3_C_2_-based colorimetric biosensor displayed a positive correlation between okadaic acid concentration and absorbance in a linearity of 10–1000 ng/mL [56].

####### Fluorescent Substances

Fluorescent substances are by far the most often used method and come in a variety of schemes for fluorescent biosensors. The principle of fluorescent biosensors relies on the fluorescence phenomenon that happens when the energy of a photon is absorbed fluorescent substances or fluorescently labeled molecules and achieves an excited state. Upon relaxation from that excited state, a fluorescent signal is emitted [57,58]. Fluorescence-based sensing is considered an evolving field of research in chemical and biochemical sciences. This technique offers some benefits such as high sensitivity, fast response, and high accuracy. Fluorescence-based biosensors have variety of schemes through employing variety of fluorimetric indicators such as organic dyes, quantum dot, and carbon dot [59,60,61]. Hu et al. constructed a fluorescence-based assay which employed carbon dots-based fluorescence microspheres (CDs@FMs) for ultrasensitive probing malachite green [62]. At free state, CDs@FMs absorbed a photon energy at 564 nm and emitted a spectrum of 581 nm. The presence of target malachite green significantly suppressed this emission pathway due to the quenching effect of malachite green. The achieved limit of detection was down to 56.7 pM. Liu et al. employed QD-loaded metal–organic framework (QD@MOF) biocomposite which was decorated with antibody (Ab) for detecting tetrodotoxin [63]. By employing the MOF property, the QD@MOF*Ab biocomposite can be synthesized via one-step and self-assembly process oriented by Zn^2+^. In these biocomposites, QD provided excellent fluorescence properties, while MOF*Ab enhanced the affinity toward tetrodotoxin. The detection limit of the QD@MOF*Ab was 0.13 ng/mL at logarithmic concentrations of 0.2–400 ng/mL. QD@MOF*Ab was also successfully used to detect tetrodotoxin in puffer fish and clam samples, demonstrating its potential for monitoring seafood. Zhou et al. used fluorescent-labeled aptamer conjugating on magnetic Fe_3_O_4_@MOF@AuNPs for detecting okadaic acid in shellfish tissue [64]. Aptamer probe can fold into three dimensional (3D) shape which mimic antibody to specifically capture okadaic acid. Upon okadaic acid binding, fluorescent-labeled aptamer was released and kept distant from Fe_3_O_4_@MOF@AuNPs, resulting in a significant increase in fluorescence intensity. By measuring fluorescence intensity, the limit of detection (LOD) and limit of quantitation (LOQ) of okadaic acid were as low as 0.015 and 0.050 ng/mL, respectively.

#### 2.2.2. SERS Biosensors

Surface-enhanced Raman scattering (SERS) is a subset of Raman scattering in which the Raman signal is enhanced by means of plasmonic metal nanostructures such as Cu-, Ag-, and Au-based nanomaterials, rendering the detection limits down to single-molecule level. SERS signals sensitively respond to an analyte when it comes close to the surface of plasmonic metal nanomaterials, also known as SERS substrates, changing the original SERS signal profile of target-free substrate. Information about the analytes is provided by measuring their Raman spectra and determining the intensity of Raman peaks. SERS biosensors offer some benefits such as: (1) provide intrinsic fingerprint molecular information of biomolecules with high sensitivity even down to single-molecule level; (2) developing alongside with nanotechnology, SERS substrates can be easily designed with different shapes and sizes to fine tune their properties providing many choices for different applications; (3) qualitative, semi-quantitative, quantitative measurement, and real-time analysis; (4) SERS biosensors allow for quick and accurate analysis in low-resource settings opposed to other sensing methods [65,66,67,68].

##### Metallic SERS Substrates

**Figure 4:**
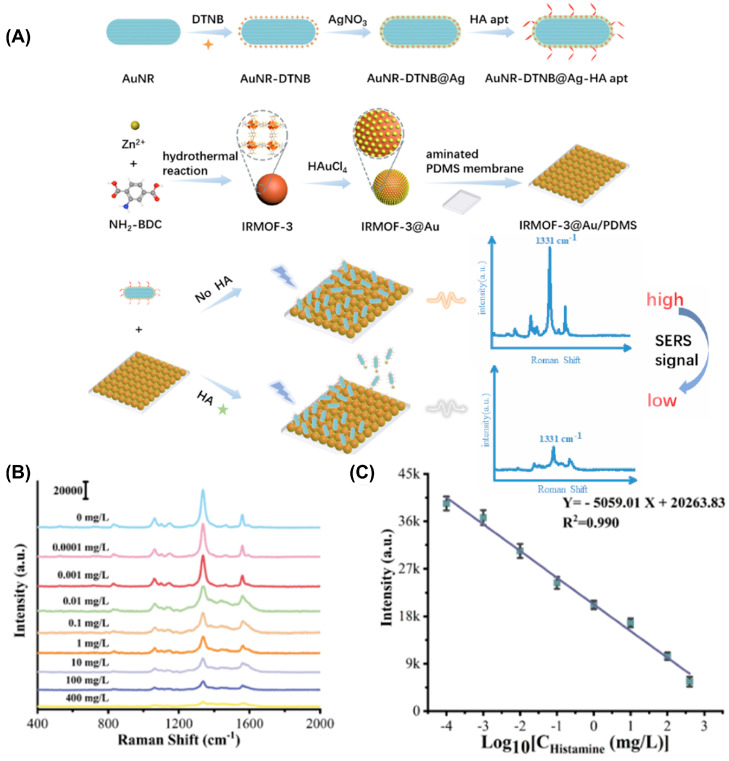
(**A**) Scheme showing the detection of histamine using SERS biosensors fabricated from metallic substrate (Au/Ag nanorod and IRMOF-3@Au/PDMS membrane). (**B**) SERS spectra of histamine at different concentrations (0.0001 mg/L to 400 mg/L). (**C**) Plot of SERS intensities at 1331 cm^−1^ as a function of the logarithmic histamine concentration [73].

There is no universal substrate that can detect all types of analytes; thus, developing different SERS substrates specifically responding to each analyte and providing different SERS signal profiles to distinguish individual analytes is important. Currently, along with significant advances in nanotechnology, a variety of composites, shapes, and sizes of SERS substrates have been synthesized with different enhancement factors. The enhancement factors directly influence the precision of SERS measurement. In seafood analysis, it is necessary to select suitable functionalized SERS substrates for more accurate target measurement. Metallic SERS substrates have excellent localized surface plasmon resonance (LSPR). Moreover, the effect of local field enhancement makes them become widely used as enhanced substrates. As a typical example, gold nanoparticles serve as promising materials for fabricating SERS substrates due to their strong LSPR and local electric field [69,70]. For example, Wei et al. used gold nanoparticles to enhance SERS signal through playing a catalytic role in the reduction process of 4-nitro thiophenol (4-NTP) to 4-aminothiophenol (4-ATP) and amplify SERS signal of the catalytic product [71]. Okadaic acid was successfully detected with a detection limit of 2.4524 ng/mL. Remarkably, gold nanomaterials can be immobilized by other materials for further improving the stability and sensitivity of SERS biosensors. For example, Guo et al. immobilized gold nanostar (AuNS) on metal–organic frameworks (MOF) [72]. MOF possessed excellent gas and matter adsorption capabilities due to their unique feature regarding 3D network of large surface area nanopores, which impressively increased the sensitivity of the biosensor. In addition, MOF acted as a sieve mesh for protecting gold nanostars from corrosion in complex testing conditions. This combination (AuNS + MOF) was used to monitor the freshness of shrimp through measuring the change in gaseous molecules and pH value. The detection ranges for gaseous molecules and pH were 10^−7^–10^−3^ (*v*/*v*) and 4–9, respectively. Pan et al. assembled flexible SERS substrate fabricated from IRMOF-3@Au/PDMS nanocomposite and Raman signal probe fabricated from AuNR-DTNB@Ag-HA aptamer (Figure 4) [73]. This complex significantly enhanced SERS signal due to synergistic effect of AuNR@Ag and IRMOF-3@Au. Meanwhile, HA aptamer enhanced the affinity of the complex toward histamine. During histamine addition, DTNB signal value on SERS substrate was decreased because histamine competitively bound to Raman signal probe. This strategy achieved a detection limit of 3.6 × 10^−5^ mg/L.

###### Colloidal and Solid SERS Substrates

Based on physical states, SERS substrates can be divided into two main categories: colloidal and solid. Noble metals such as gold and silver nanoparticles with monodispersion and particle diameters of 10–200 nm are commonly used to generate colloidal SERS substrates. Colloidal SERS substrates are highly sensitive, have simple fabrication and liquid-flow capacity, thus, they are widely applied for fabricating SERS biosensors [74]. As mentioned above, the SERS signal intensity of substrates is highly influenced by the shape and size of metal particles [75]. Zhou et al. demonstrated that with the increased sizes of silver triangle nanoparticles (AgTNP), the SERS intensity of AgTNPs decreased. Particularly, small silver nanoparticles with triangle shape had higher ratios of the side faces to the top and bottom surfaces than that of large AgTNP, suggesting that small AgTNP could capture more probe molecules. Moreover, the hotspot located at the tips and the probe molecules of small AgTNP were closer than those of large AgTNP because of their smaller volume [76]. Li et al. used colloidal gold nanoparticles for detecting histamine through SERS analysis [77]. Histamine reacted with o-phthalaldehyde to generate a Schiff base product (O-His) resulting in a change in SERS activities. In this strategy, gold nanoparticles displayed an important role in increasing SERS signal. Under the optimized conditions, this SERS system could produce SERS intensity which was linearly proportional to the histamine concentration in the range of 0.05–4.5 mg/L with a detection limit of 0.04 mg/L. This system was also successfully used to detect histamine in seafood.

However, colloidal SERS substrates generally witness some limitations. First, metal nanoparticles in liquid solution are not stable due to the electrostatic repulsion existing between the metal nanoparticles. The electrostatic repulsion represents the reason for aggregation of metal nanoparticles over time. Thus, colloidal SERS substrates meet a critical challenge regarding their stability over time. Second, colloidal SERS substrates are a liquid system, it is extremely difficult to determine the position of the target analytes. Third, the colloidal state is highly influenced by pH value; thus, the pH of sample can significantly affect the colloidal SERS substrates.

To overcome the limitations of colloidal SERS substrates, scientists immobilize metal nanoparticles onto solid substrates. In the past few years, solid SERS substrates have been impressively developed due to their good properties such as high sensitivity, good stability, and convenient portability. Moreover, solid SERS substrates show excellent features in several aspects. First, there are a variety of methods for the preparation of solid SERS substrates such as spin coating, self-assembly method, nanolithography, filtration, chemical vapor deposition. Second, solid SERS substrates possess significantly high SERS signals due to highly dense hot spots originating from concentrated unit area. Third, uniform, stable, and reproducible SERS signals can be obtained through controlling morphology and structure. Fourth, the solid nature of substrates significantly increases the stability of metal nanoparticles immobilized on them, expanding the scope of SERS analysis in practical applications. There are a variety of solid materials that can be used for immobilizing SERS active elements such as glass, elastomer, paper, membrane, plasmonic film, and metal alloy. Das et al. used a smart container which employed glass Petri dish as solid substrate for immobilizing gold nanosphere (AuNS@Dish) [78]. It was demonstrated that the AuNS@Dish served as an efficient plasmonic SERS substrate with an analytical enhancement factor (AEF) of 10^6^. The AuNS@Dish was used to determine indole level which is an important parameter to monitoring bacterial contamination in seafood. Upon adding indole-containing shrimp extract on the AuNS@Dish, SERS signal was strongly increased because of the high AEF of the prepared AuNS@Dish. The AuNS@Dish could detect indole as low as 0.009 μg/100 g of shrimp (the FDA regulated level = 25 μg indole/100 g of shrimp). Slippery liquid-infused porous surface (SLIPS) had been exploited for fabricating solid SERS substrate by Guo et al. due to its properties such as defect-free, stable, and inert slippery interface [72]. The SLIPS-based SERS biosensor was fabricated by dropping AuNS@ZIF-8 on the SLIPS substrate generating ellipse-like aggregations without coffee ring effect. This droplet acted as SERS sensor providing enhanced SERS signals in the presence of target analytes. Table 1 shows the summary of biosensors for monitoring seafood.

Generally, SERS biosensors displayed satisfactory sensitivity with a broad linear range. SERS biosensor that was developed from Fe_3_O_4_ magnetic microspheres surrounded by gold-Prussian blue-Au nanoparticles (FAPANPs), possessed a low limit of detection (0.0027 ng/mL) with a great linearity of 0.01–500 ng/mL for tetrodotoxin detection [79]. Wu et al. used an HAuCl_4_/K_4_Fe(CN)_6_ reaction-mediated silver nanosol as SERS substrate for detection of adenine, ceftriaxone, and malachite green in seafood [80]. This SERS biosensor possessed linear ranges of 0.1–20 μM, 0.1–10 μM, and 0.1–2 μM for detecting adenine, ceftriaxone, and malachite green, respectively. Adade et al. detected benzo(*b*)fluoranthene (a carcinogenic contaminant in seafood) with a strong linear relationship (R^2^ = 0.9934) within the range of 0.4 to 12.9 ng/mL [81].

**Table 1:** Summary of biosensors for monitoring seafood.

Biosensors and Materials	Application	LOD	Target	Type of Sample
Electrochemical biosensor, carbon black modified SPEs [29]	Detect marine toxin	0.18 ng/mL	Okadaic acid	Mussel extract
Electrochemical biosensor, 2D carbon nitride-aptamer-based electrode [82]	Detect marine toxin	0.08 pg/mL	Okadaic acid	Shellfish samples
Electrochemical biosensor, K_3_Fe(CN)_6_ regulated Ag NPs@Apt [83]	Detect marine toxin	1 nM	Saxitoxin (STX)	Clams, Mantis shrimps
Electrochemical biosensor, MIP sensor [39]	Detect marine toxin	1.14 μg/mL	Tetrodotoxin	Mussel samples
Electrochemical biosensor, MoS_2_-PLL-Apt electrode [27]	Detect foodborne pathogen	5.74 CFU/mL	*Vibrio parahaemolyticus*	Shrimp food samples
Electrochemical biosensor, apt-AuNP@NH_2_-VMSF/PDA/GCE [28]	Detect foodborne pathogen	10^3^ CFU/mL	*Vibrio parahaemolyticus*	Marine shrimp
Electrochemical biosensor, HIROF/SPE electrode [84]	Detect foodborne pathogen	10^3^ CFU/mL	*Vibrio parahaemolyticus*	Fish samples
Electrochemical biosensor, COOH-MWCNTs-Fe_3_O_4_-GO nanohybrids [85]	Detect antimicrobial	0.003 ng/mL	Sulfadimidine	Crayfish
Electrochemical biosensor, PDA@ZnMoO_4_/MXene composite [17]	Detect foodborne pathogen	12 CFU/mL	*Listeria monocytogenes*	Smoked seafood
Electrochemical biosensor, magnetic-MIP [33]	Monitor seafood freshness	1.6 × 10^−6^ mg/L	Scombrotoxin (histamine)	Fish samples
Colorimetric biosensor, CDs@FMs probe [62]	Detect residues of antifungal agent	56.7 pM	Malachite green	Fish samples
Colorimetric biosensor, BTSIXO [86]	Detect Endocrine Disrupting Chemicals (EDC)	0.02 ppm	Bisphenol A	Fish samples
Colorimetric biosensor, magnetic bead IgG-HRP [87]	Detect neurotoxin	1 μg/kg (oyster and razor clam samples) 3.3 μg/kg (mussel samples)	Tetrodotoxin (TTX)	Pacific oysters Razor clams Mussels
Colorimetric biosensor, QD@MOF*Ab probes [63]	Detect neurotoxin	0.4 ng/mL	Tetrodotoxin (TTX)	Fishes Clams
Colorimetric biosensor, Au@Pt NPs/horseradish peroxidase [53]	Detect marine toxin	0.6 µg/kg (mussel tissues)	Okadaic acid	Oysters Mussels Clams
Colorimetric biosensor, Fe_3_O_4_@MOF@AuNPs [64]	Detect marine toxin	0.015 ng/mL	Okadaic acid	Shellfish samples
Colorimetric biosensor, CeO_2_@PtRu nanozyme [52]	Detect foodborne pathogen	193 CFU/mL	*Vibrio vulnificus*	Clams, Shrimps
Colorimetric biosensor, *E. amoenum* extract [88]	Monitor seafood freshness	Respond to pH by changing color from red to yellow over the pH range of 2–12	pH value	Shrimp samples
Colorimetric biosensor, self-assembled polydiacetylene [89]	Monitor seafood freshness	70 ppm	Histamine	Spanish mackerel, Tuna, Mackerel
Colorimetric biosensor, Au^0^-NPs_ALz_ [90]	Monitor seafood freshness	59.32 μmol/L	Histamine	White shrimp, giant tiger prawn, cuttlefish, and splendid squid
Colorimetric biosensor, CF/CNF_10_/SSA [91]	Monitor seafood freshness	Respond to pH by changing color from red to yellow over the pH range of 1–12	Ammonia and pH	Shrimp
SERS biosensor, heterogeneous nano pineapples [92]	Detect residues of antifungal agent	7.8 × 10^−11^ M	Malachite green	Clams
SERS biosensor, Ag TNP@SiO_2_ [76]	Detect residues of antifungal agent	0.49 pM	Malachite green	Spiked water
SERS biosensor, HAuCl_4_/K_4_Fe(CN)_6_ reaction mediated silver nanosol [80]	Detect residues of antifungal agent	0.032 μM	Malachite green	Tilapia, shrimps
SERS biosensor, aptamer-recognized SERS tag [93]	Detect toxin	0.1 ng/L	Microcystin-LR (MC-LR)	Fish organs
SERS biosensor, GO-Au [94]	Detect marine toxin	5.47 nM	Stonehouse clam toxin	Clam muscle tissue
SERS biosensor, Fe_3_O_4_@MOF-GNS-MBA-Apt [95]	Detect foodborne pathogen	7 CFU/mL	*Vibrio parahaemolyticus*	Shrimps
SERS biosensor, β-CD-AgNPs [96]	Monitor seafood freshness	7.2 nM	Histamine	Fishes

## 3. Conclusions and Future Perspectives

This review summarized current reports on biosensors for seafood monitoring. Along with significant advances in biosensors, the processes of seafood monitoring can be simplified and performed outside the laboratory. Biosensors provide the simultaneous detection of multiple target analytes which allows multiple analysis in single operation. Biosensors offer several advantages over basic laboratory tests such as higher sensitivity, selectivity, and portability. In this review, we summarized two main types of biosensors including electrochemical and optical biosensors (colorimetric and SERS biosensors) and discussed their working mechanisms and benefits. Although these biosensors showed significant advantages over conventional laboratory methods, each type of biosensor has its own limitations which future research should focus on to improve them.

Electrochemical biosensors provide a highly sensitive approach for monitoring seafood safety; however, they require external electrical power and a potentiostat. This electrical equipment limits the application of electrochemical biosensors in low-resource environments. In addition, the result output is displayed as the electric current which might be difficult for the general users.

Unlike electrochemical biosensors, colorimetric biosensors do not require bulky electrical equipment for operation. They provide user-friendly result readouts in which the negative and positive samples can be simply distinguished based on the difference in color. However, the colorimetric biosensors still witness some limitations. First, colorimetric biosensors require biochemical reactions with multiple chemical reagents. Some of these reagents require special storage conditions. Second, the operation of colorimetric biosensors comprises a series of handling steps for adding and mixing liquid reagents which might increase difficulty for non-expert users.

SERS biosensors undergo significant progress in the current decades due to the impressive development of nanomaterials which increases the sensitivity, selectivity, and stability of SERS biosensors. However, SERS biosensors still need further improvements. In the near future, research on SERS biosensors should focus on finding a solution to omit the use of bulky equipment such as laser systems and Raman signal detectors.
